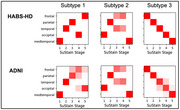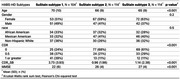# Assessing the Generalizability of ADRDs Subtypes Across Diverse Cohorts HABS‐HD and ADNI

**DOI:** 10.1002/alz70856_107005

**Published:** 2026-01-07

**Authors:** Gordon Zhaoqi An, Brian A. Gordon, Aristeidis Sotiras, Peter R Millar, Beau Ances, Sid E. O'Bryant, Karin L. Meeker

**Affiliations:** ^1^ Division of Computational and Data Sciences, Washington University in St. Louis, St. Louis, MO, USA; ^2^ Mallinckrodt Institute of Radiology, Washington University School of Medicine, St. Louis, MO, USA; ^3^ Institute for Informatics, Washington University, St. Louis, MO, USA; ^4^ Mallinckrodt Institute of Radiology, Washington University School of Medicine in St Louis, St Louis, MO, USA; ^5^ Washington University School of Medicine, St. Louis, MO, USA; ^6^ Washington University at St. Louis, St. Louis, MO, USA; ^7^ Institute for Translational Research, University of North Texas Health Science Center, Fort Worth, TX, USA

## Abstract

**Background:**

Alzheimer's disease and related dementias (ADRDs) presents significant biological heterogeneity, which influences clinical outcomes and treatment responses. However, current ADRDs research has been predominately conducted in non‐Hispanic white cohorts, such as the Alzheimer's Disease Neuroimaging Initiative (ADNI), limiting the understanding of ADRDs progression in diverse populations. The Health and Aging Brain Study – Health Disparities (HABS‐HD), which includes Black/African American (AA), Hispanic (HIS), and non‐Hispanic white (NHW) participants, offers a broader sociodemographic perspective. This study aims to test the generalizability of ADRDs subtypes and progression patterns identified in ADNI to the more diverse HABS‐HD cohort.

**Method:**

Structural MRI data from HABS‐HD (AA *n* = 588, HIS *n* = 1005, NHW *n* = 1009) and ADNI (*n* = 864) cohorts were processed using FreeSurfer to extract brain cortical thickness and hippocampal volume. The Subtype and Stage Inference (SuStaIn) algorithm was then applied to both datasets to identify spatial atrophy subtypes and disease stages. To compare subtypes between cohorts, Positional Variance Diagrams (PVDs) in Figure 1. were used to visualize the variability and uncertainty in disease progression patterns derived by the model.

**Result:**

SuStaIn identified three spatial atrophy subtypes and progression stages in HABS‐HD and ADNI. HABS‐HD's PVD patterns aligned with ADNI's, revealing distinct progression patterns: Subtype 1 was characterized by initial degeneration of the hippocampus, followed by posterior and then anterior spread; Subtype 2 began with occipital atrophy, progressing to parietal regions; Subtype 3 was characterized by initial frontal degeneration, with subsequent spread to posterior regions. The table 1. Shows significant differences in HABS‐HD's distinct demographic and clinical features (*p* < 0.05), where Subtype 3 exhibits the most severe cognitive impairment and represents the oldest age group.

**Conclusion:**

Similarity of disease progression patterns between HABS‐HD and ADNI supports generalizability of AD subtypes to diverse populations, highlighting the subtypes' potential to advance clinical trials and precision medicine for neurodegenerative disorders.